# Supplementary material for: Modeling glioblastoma heterogeneity as a dynamic network of cell states
Source: Mol Syst Biol. 2021 Sep 16;17(9):e10105. doi: 10.15252/msb.202010105 (PMC8444284; doi:10.15252/msb.202010105)
Supplement: Supplementary file 5 — Source Data for Figure 3 [file MSB-17-e10105-s001.zip › Figure3A_sourcedata/GSEA_3065/nosigdb_state1.GseaPreranked.1622718409535/NB_KI_CLUSTER16.html]

Details for gene set NB\_KI\_CLUSTER16[GSEA]

|  || Dataset | state1 |
| Phenotype | NoPhenotypeAvailable |
| Upregulated in class | na\_pos |
| GeneSet | NB\_KI\_CLUSTER16 |
| Enrichment Score (ES) | 0.53721243 |
| Normalized Enrichment Score (NES) | 1.855537 |
| Nominal p-value | 0.0 |
| FDR q-value | 0.0010875412 |
| FWER p-Value | 0.023 |
Table: GSEA Results Summary

  

Fig 1: Enrichment plot: NB\_KI\_CLUSTER16      
 Profile of the Running ES Score & Positions of GeneSet Members on the Rank Ordered List

  

| PROBE | GENE SYMBOL | GENE\_TITLE | RANK IN GENE LIST | RANK METRIC SCORE | RUNNING ES | CORE ENRICHMENT || 1 | CSRP2 |  |  | 28 | 0.574 | 0.0443 | Yes |
| 2 | MAP3K7CL |  |  | 32 | 0.546 | 0.0888 | Yes |
| 3 | FBXO32 |  |  | 39 | 0.523 | 0.1311 | Yes |
| 4 | MYL6 |  |  | 68 | 0.420 | 0.1627 | Yes |
| 5 | DSTN |  |  | 70 | 0.417 | 0.1968 | Yes |
| 6 | LDHA |  |  | 80 | 0.394 | 0.2282 | Yes |
| 7 | ACTB |  |  | 86 | 0.386 | 0.2594 | Yes |
| 8 | ARHGAP29 |  |  | 120 | 0.346 | 0.2844 | Yes |
| 9 | TPM1 |  |  | 132 | 0.335 | 0.3108 | Yes |
| 10 | FHL2 |  |  | 141 | 0.328 | 0.3368 | Yes |
| 11 | NRGN |  |  | 204 | 0.294 | 0.3546 | Yes |
| 12 | ACTN1 |  |  | 230 | 0.284 | 0.3754 | Yes |
| 13 | SLC25A5 |  |  | 254 | 0.276 | 0.3957 | Yes |
| 14 | TACC1 |  |  | 258 | 0.275 | 0.4180 | Yes |
| 15 | PTMA |  |  | 366 | 0.236 | 0.4264 | Yes |
| 16 | TM4SF1 |  |  | 379 | 0.231 | 0.4442 | Yes |
| 17 | LBH |  |  | 435 | 0.220 | 0.4566 | Yes |
| 18 | CSRP1 |  |  | 504 | 0.205 | 0.4665 | Yes |
| 19 | HSPB1 |  |  | 531 | 0.199 | 0.4802 | Yes |
| 20 | EPS8 |  |  | 571 | 0.190 | 0.4918 | Yes |
| 21 | TGFB1I1 |  |  | 580 | 0.189 | 0.5065 | Yes |
| 22 | MGLL |  |  | 633 | 0.181 | 0.5161 | Yes |
| 23 | PALLD |  |  | 646 | 0.179 | 0.5296 | Yes |
| 24 | TSC22D1 |  |  | 881 | 0.147 | 0.5177 | Yes |
| 25 | CAV2 |  |  | 1146 | 0.118 | 0.5005 | Yes |
| 26 | PHLDB2 |  |  | 1225 | 0.110 | 0.5016 | Yes |
| 27 | GAS6 |  |  | 1253 | 0.108 | 0.5077 | Yes |
| 28 | VASP |  |  | 1280 | 0.106 | 0.5137 | Yes |
| 29 | SELENOW |  |  | 1298 | 0.103 | 0.5205 | Yes |
| 30 | TLN1 |  |  | 1341 | 0.100 | 0.5244 | Yes |
| 31 | RBPMS |  |  | 1404 | 0.095 | 0.5258 | Yes |
| 32 | KIAA0040 |  |  | 1443 | 0.093 | 0.5296 | Yes |
| 33 | ANGPT2 |  |  | 1496 | 0.089 | 0.5316 | Yes |
| 34 | KANK2 |  |  | 1513 | 0.088 | 0.5372 | Yes |
| 35 | CRIM1 |  |  | 1632 | 0.080 | 0.5317 | No |
| 36 | SEPT4 |  |  | 1732 | 0.073 | 0.5277 | No |
| 37 | ACTA2 |  |  | 1800 | 0.069 | 0.5265 | No |
| 38 | ARHGAP10 |  |  | 1969 | 0.061 | 0.5144 | No |
| 39 | ADAMTS9 |  |  | 2489 | 0.040 | 0.4647 | No |
| 40 | SYTL2 |  |  | 2522 | 0.039 | 0.4647 | No |
| 41 | CPED1 |  |  | 2606 | 0.036 | 0.4592 | No |
| 42 | INPP4B |  |  | 2636 | 0.035 | 0.4591 | No |
| 43 | PAWR |  |  | 2666 | 0.034 | 0.4589 | No |
| 44 | GEM |  |  | 2691 | 0.033 | 0.4592 | No |
| 45 | ATF3 |  |  | 2855 | 0.029 | 0.4449 | No |
| 46 | ARID5A |  |  | 2910 | 0.027 | 0.4416 | No |
| 47 | UTRN |  |  | 2994 | 0.025 | 0.4352 | No |
| 48 | MIR22HG |  |  | 3494 | 0.014 | 0.3854 | No |
| 49 | EPAS1 |  |  | 3624 | 0.011 | 0.3732 | No |
| 50 | SMTN |  |  | 3761 | 0.009 | 0.3600 | No |
| 51 | SORBS3 |  |  | 3765 | 0.009 | 0.3605 | No |
| 52 | CCDC102B |  |  | 3847 | 0.007 | 0.3528 | No |
| 53 | ACTN4 |  |  | 3909 | 0.006 | 0.3471 | No |
| 54 | MYC |  |  | 3964 | 0.005 | 0.3420 | No |
| 55 | COBLL1 |  |  | 4045 | 0.003 | 0.3341 | No |
| 56 | CCDC107 |  |  | 4067 | 0.003 | 0.3322 | No |
| 57 | OAZ2 |  |  | 4471 | -0.004 | 0.2914 | No |
| 58 | ADCY3 |  |  | 5714 | -0.024 | 0.1666 | No |
| 59 | MT1M |  |  | 5737 | -0.024 | 0.1663 | No |
| 60 | PPP1R12A |  |  | 5825 | -0.026 | 0.1596 | No |
| 61 | MAP7D3 |  |  | 6158 | -0.032 | 0.1283 | No |
| 62 | PLEKHG2 |  |  | 6392 | -0.036 | 0.1075 | No |
| 63 | ID1 |  |  | 6749 | -0.044 | 0.0748 | No |
| 64 | DMPK |  |  | 6879 | -0.047 | 0.0654 | No |
| 65 | FRZB |  |  | 7032 | -0.051 | 0.0541 | No |
| 66 | MAP3K20 |  |  | 7115 | -0.054 | 0.0501 | No |
| 67 | PTK2 |  |  | 7170 | -0.055 | 0.0491 | No |
| 68 | JAG1 |  |  | 7426 | -0.061 | 0.0281 | No |
| 69 | BCAM |  |  | 7587 | -0.066 | 0.0172 | No |
| 70 | LPP |  |  | 8255 | -0.092 | -0.0433 | No |
| 71 | PDLIM3 |  |  | 8278 | -0.093 | -0.0379 | No |
| 72 | ISYNA1 |  |  | 8434 | -0.101 | -0.0454 | No |
| 73 | PEAK1 |  |  | 8607 | -0.112 | -0.0538 | No |
| 74 | CPE |  |  | 9147 | -0.157 | -0.0958 | No |
| 75 | IGFBP2 |  |  | 9209 | -0.167 | -0.0884 | No |
| 76 | PDGFA |  |  | 9341 | -0.188 | -0.0863 | No |
| 77 | MCAM |  |  | 9468 | -0.218 | -0.0813 | No |
| 78 | SPECC1 |  |  | 9470 | -0.219 | -0.0634 | No |
| 79 | MIDN |  |  | 9590 | -0.256 | -0.0545 | No |
| 80 | NUDT4 |  |  | 9630 | -0.272 | -0.0362 | No |
| 81 | SEMA5A |  |  | 9860 | -0.749 | 0.0019 | No |
Table: GSEA details [plain text format]

  

Fig 2: NB\_KI\_CLUSTER16: Random ES distribution      
 Gene set null distribution of ES for **NB\_KI\_CLUSTER16**
